# Autocrine Human Growth Hormone Promotes Invasive and Cancer Stem Cell-Like Behavior of Hepatocellular Carcinoma Cells by STAT3 Dependent Inhibition of CLAUDIN-1 Expression

**DOI:** 10.3390/ijms18061274

**Published:** 2017-06-15

**Authors:** Yi-Jun Chen, Ming-Liang You, Qing-Yun Chong, Vijay Pandey, Qiu-Shi Zhuang, Dong-Xu Liu, Lan Ma, Tao Zhu, Peter E. Lobie

**Affiliations:** 1 Cancer Science Institute of Singapore and Department of Pharmacology, National University of Singapore, Singapore 119077, Singapore; yjchen011@gmail.com (Y.-J.C.); csiym@nus.edu.sg (M.-L.Y.); csicqy@nus.edu.sg (Q.-Y.C.); csivkp@nus.edu.sg (V.P.); zhuangqiushi2007@yahoo.com (Q.-S.Z.); 2 School of Science, Faculty of Health and Environmental Sciences, Auckland University of Technology, Auckland 1010, New Zealand; dong-xu.liu@aut.ac.nz; 3 Tsinghua Berkeley Shenzhen Institute (TBSI), Graduate School at Shenzhen, Tsinghua University, Shenzhen 518055, China; malan@sz.tsinghua.edu.cn; 4 Hefei National Laboratory for Physical Sciences at Microscale, University of Science and Technology of China, Hefei 230026, China; zhut@ustc.edu.cn; 5 The CAS Key Laboratory of Innate Immunity and Chronic Disease, School of Life Sciences and Medical Center, University of Science and Technology of China, Hefei 230022, China

**Keywords:** human growth hormone, hepatocellular carcinoma, STAT3, CLAUDIN-1, cancer stem cells

## Abstract

Despite progress in diagnosis and treatment of hepatocellular carcinoma (HCC), the clinical outcome is still unsatisfactory. Increased expression of human growth hormone (hGH) in HCC has been reported and is associated with poor survival outcome in HCC patients. Herein, we investigated the mechanism of the oncogenic effects of hGH in HCC cell lines. In vitro functional assays demonstrated that forced expression of hGH in these HCC cell lines promoted cell proliferation, cell survival, anchorage-independent growth, cell migration, and invasion, as previously reported. In addition, forced expression of hGH promoted cancer stem cell (CSC)-like properties of HCC cells. The increased invasive and CSC-like properties of HCC cells with forced expression of hGH were mediated by inhibition of the expression of the tight junction component CLAUDIN-1. Consistently, depletion of CLAUDIN-1 expression increased the invasive and CSC-like properties of HCC cell lines. Moreover, forced expression of CLAUDIN-1 abrogated the acquired invasive and CSC-like properties of HCC cell lines with forced expression of hGH. We further demonstrated that forced expression of hGH inhibited CLAUDIN-1 expression in HCC cell lines via signal transducer and activator of transcription 3 (STAT3) mediated inhibition of CLAUDIN-1 transcription. Hence, we have elucidated a novel hGH-STAT3-CLAUDIN-1 axis responsible for invasive and CSC-like properties in HCC. Inhibition of hGH should be considered as a therapeutic option to hinder progression and relapse of HCC.

## 1. Introduction

Hepatocellular carcinoma (HCC) makes up 85–90% of primary liver cancer [1]. Despite advances in the diagnosis and treatment of HCC, mortality rate remains high due to frequent recurrence and intrahepatic metastasis [2]. Recently, the identification of cancer stem cells (CSCs) or tumor initiating cells in HCC further dampens the current routine treatments including chemotherapy, and underscores the necessity for novel therapeutic strategies [3,4].

The liver is an essential target tissue of pituitary human growth hormone (hGH) to exert pleiotropic effects on postnatal somatic growth and metabolism processes through stimulation of hepatic insulin-like growth factor-1 (IGF-1) [5]. The relationship between endocrine growth hormone (GH) and cancer has been widely reported in human and animal models [6,7,8]. In addition to functioning as a hormone, hGH also acts at an autocrine or paracrine level [9]. The oncogenic capacity of autocrine hGH has been intensively investigated in human breast and endometrial cancer cells [10,11]. Autocrine hGH has been involved in promoting tumorigenic behaviors of breast and endometrial cancer cells, including epithelial-mesenchymal transition (EMT) [12,13] and cancer stem cell (CSC)-like properties [14]. The autocrine hGH-stimulated EMT phenotypic conversion in breast cancer cells was reported to be partially mediated by signal transducer and activator of transcription 3 (STAT3) activation [13], which also mediated the oncogenicity of autocrine hGH in endometrial cancer cells [15].

Aberrations in GH/GHR axis have previously been implicated in HCC development. GH transgenic mice exhibited increased incidence of spontaneous and carcinogen-induced HCC development [16,17]. Conversely, GH-deficient lit/lit mice exhibited reduced susceptibility to carcinogen-induced hepatocarcinogenesis [18]. Whilst an earlier study reported loss of the hGH receptor (hGHR) in HCC, another study has demonstrated the increased expression of hGHR at the cellular levels in HCC samples, compared to normal liver tissues [19]. Moreover, we have recently observed increased expression of *hGH* mRNA in HCC patient samples in comparison to the paired normal liver tissues [20]. In addition, tumor expression of hGH mRNA was associated with poor relapse-free survival (RFS) and overall survival (OS) outcomes in a cohort of HCC patients [20]. Forced expression of hGH was demonstrated to promote cell proliferation, survival and invasion of HCC cells through the activation of STAT3 in vitro [20]. Concordantly, autocrine hGH promoted growth of HCC cell generated xenografts [20]. However, the underlying mechanism of autocrine hGH-mediated HCC progression has yet to be elucidated.

CLAUDIN-1 is a member of the CLAUDIN (CLDN) family, consisting of 27 tetraspan transmembrane proteins expressed in a tissue-specific pattern [21]. They are important constituents of tight junctions, where they establish the paracellular barrier and maintain the cellular polarity [21]. More recently, studies have demonstrated that the tight junction proteins are involved in cellular signal transduction affecting cell proliferation, motility, and invasion [22]. Aberrant expression of CLDNs has been observed in diverse types of human cancers, including HCC [21]. CLAUDIN-1 exhibits tissue specific effects on cancer progression. Although low CLAUDIN-1 expression has been reported to independently predict for poor clinical outcome in colon cancer patients [23], in vitro and in vivo studies have demonstrated that CLAUDIN-1 promotes EMT conversion of colon cancer cells through zinc finger E-box-binding homeobox 1 (ZEB-1) mediated inhibition of E-cadherin expression [24,25]. Conversely, CLAUDIN-1 exhibited tumor suppressive activity and mediated the tumor suppressor function of transcription factor RUNX3 in gastric cancer cells [26]. Immunohistochemical investigations have identified attenuated expression of CLAUDIN-1 as a potential marker for poor prognosis in poorly differentiated HCC [27], suggestive of tumor suppressive effects of CLAUDIN-1 in HCC.

In the current study, we observed that autocrine hGH promoted HCC cell invasion and CSC-like properties. We further demonstrated that autocrine hGH promotion of cancer progression in HCC cells was mediated by STAT3 dependent inhibition of CLAUDIN-1 expression.

## 2. Results

### 2.1. Forced Expression of Human Growth Hormone (hGH) Promotes Monolayer, Anchorage-Independent and Three-Dimensional (3D) Matrigel Growth of Human Hepatocellular Carcinoma (HCC) Cells, and Protects Human HCC Cells from Apoptosis

Before investigating the functional effects of hGH in HCC cells, we first determined the expression of human growth hormone receptor (hGHR) and human prolactin receptor (hPRLR) expression by reverse transcription polymerase chain reaction (RT-PCR) in several HCC cell lines, including a normal hepatic cell line LO2 and a hepatoma cell line HepG2. All of these cell lines expressed detectable level of *hGHR* mRNA and *hPRLR* mRNA, except LO2 cells, which did not express detectable levels of *hPRLR* mRNA (Figure S1A).

**Figure 1:**
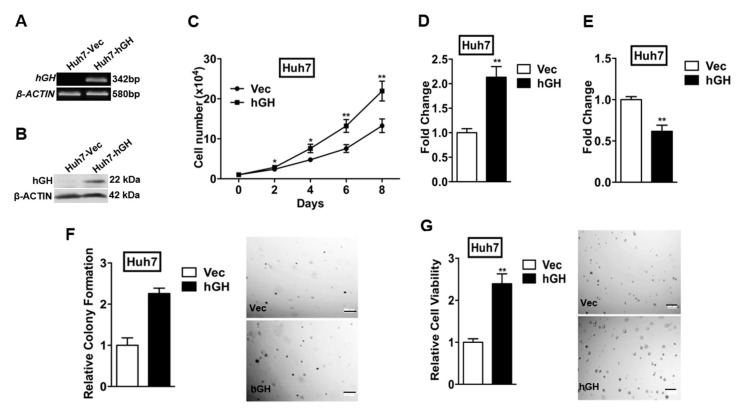
Forced expression of hGH promotes cell proliferation, cell survival, and anchorage-independent growth in human HCC cells. Huh7 cells were stably transfected with an expression vector containing the human growth hormone (*hGH*) gene (designated Huh7-hGH) or pcDNA vector alone (Huh7-Vec). The expression levels of hGH in Huh7-hGH and Huh7-Vec cell lines were determined by: (**A**) reverse transcription polymerase chain reaction (RT-PCR), and (**B**) Western blot. β-ACTIN was used as input control; (**C**) Growth of Huh7-Vec and Huh7-hGH cells were determined by total cell number assay in medium supplemented with 10% fetal bovine serum (FBS). * *p* < 0.05; ** *p* < 0.01; (**D**) Effect of forced expression of hGH on cell cycle progression was assessed by 5-bromo-2′-deoxyuridine (BrdU) incorporation in medium supplemented with 10% FBS; (**E**) Effect of forced expression of hGH on cell apoptosis was determined by measuring caspase 3/7 activity of Huh7-Vec and Huh7-hGH cell lines after 24 h serum-deprivation. ** *p* < 0.01; (**F**) The anchorage independent growth of Huh7 cells by forced expression of hGH was assessed by soft agar colony formation in 10% FBS medium. Cell colonies were visualized by 3-(4,5-dimethylthiazol-2-yl)-2,5-diphenyltetrazolium bromide (MTT) staining. Bar, 200 µm; ** *p* < 0.01; (**G**) Effect of forced expression of hGH on the growth of Huh7 cells on growth factor-reduced Matrigel™. Huh7-Vec/hGH cells were seeded in 4% Matrigel™ into 96-well plates pre-coated with Matrigel™. Cell viability was measured using MTT assay after 7 days. Bar, 200 µm; ** *p* < 0.01.

Autocrine hGH has previously been demonstrated to promote the oncogenic properties of human mammary and endometrial carcinoma cells [11,28] and also in the HCC cell line Bel-7404 and hepatoma cell line HepG2 [20]. To further determine the functional roles of autocrine hGH in HCC cells, two different HCC cell lines (Huh7 and Hep3B) and one hepatoma cell line (HepG2) were stably transfected with the hGH expression vector (designated as Huh7-hGH, Hep3B-hGH, and HepG2-hGH cells) or the empty pcDNA3.1 vector (designated as Huh7-Vec, Hep3B-Vec, and HepG2-Vec cells). Expression of hGH mRNA and protein in stably transfected Huh7, Hep3B and HepG2 cells was verified by RT-PCR and Western blot analysis, respectively (Figure 1A,B and Figure S2A,B).

To verify the functionality of forced expression of hGH in HCC cells, and as previously reported [20], we first determined the effects of forced expression of hGH on total cell number, cell cycle progression, and apoptosis in monolayer culture. The total cell number of Huh7-hGH cells increased dramatically more than Huh7-Vec cells over a period of eight days in 10% serum conditions (Figure 1C). Increases in total cell number were also observed in HepG2-hGH cells compared with HepG2-Vec cells in 10% serum conditions (Figure S2C). Increased cell number can be attributed to the net effect of increased cell proliferation and/or decreased cell apoptosis. 5-bromo-2′-deoxyuridine (BrdU) incorporation assays revealed that forced expression of hGH significantly increased cell cycle progression of Huh7 and HepG2 cells in 10% serum conditions (Figure 1D and Figure S2D). In addition, forced expression of hGH significantly reduced Huh7 and HepG2 cell apoptosis consequent to serum deprivation as indicated by a decrease in caspase 3/7 activities in the hGH cells as compared to the Vec cells (Figure 1E and Figure S2E).

We next examined the effect of forced expression of hGH on anchorage-independent growth, a hallmark characteristic of oncogenic transformation [11]. Forced expression of hGH in Huh7-hGH cells significantly enhanced anchorage-independent growth as indicated by increased colony formation in soft agar, compared with Huh7-Vec cells (Figure 1F). The same effect of increased soft agar colony formation upon forced expression of hGH was also observed in HepG2 cells (Figure S2F). We also determined the three-dimensional growth of Huh7 and HepG2 cells in the Matrigel (basement membrane extract) and demonstrated that Huh7-hGH and HepG2-hGH cells formed increased number and larger sized colonies in 3D Matrigel as compared to those of Huh7-Vec and HepG2-Vec cells respectively (Figure 1G and Figure S2G).

### 2.2. Forced Expression of hGH Promotes Migration and Invasion of HCC Cells

**Figure 2:**
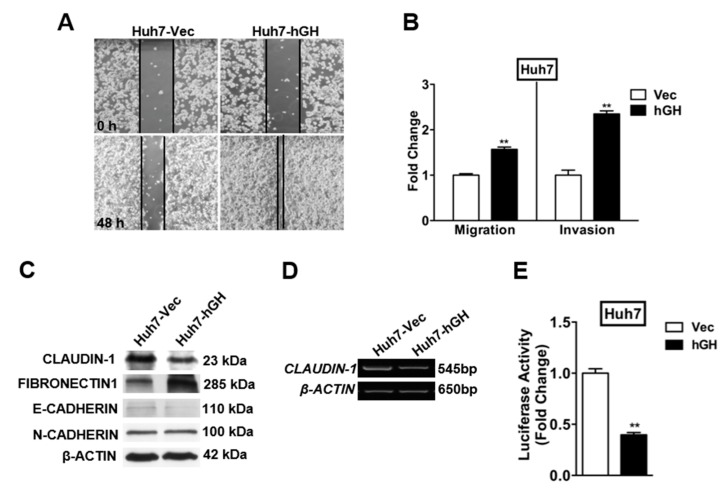
Forced expression of hGH promotes cell motility and an invasive phenotype in human HCC cells. (**A**) Cell motility was assessed by wound-healing assay under 100× magnification; (**B**) The effect of forced expression of hGH on Huh7 cell migration and invasion was determined by Transwell assay, ** *p* < 0.01; (**C**) Western blot analysis of the expression of epithelial and mesenchymal markers in Huh7-Vec/hGH cell lines; (**D**) The mRNA expression of *CLAUDIN-1* in Huh7-Vec/hGH cell lines was assessed by RT-PCR; (**E**) CLAUDIN-1 promoter activity in Huh7-Vec/hGH cell lines was assessed by luciferase reporter assay, ** *p* < 0.01.

During carcinoma progression toward a less differentiated and more invasive state, cells convert from an epithelial to a mesenchymal phenotype, accompanied with changes in gene expression, referred to as EMT [29]. Previous studies have demonstrated that forced expression of hGH promotes migration and invasion of human mammary, endometrial carcinoma cells and HCC cells by inducing EMT [11,12,20]. Using a wound-healing assay to examine cell motility in this study, we observed that forced expression of hGH stimulated Huh7 and HepG2 cell migration with more rapid wound closure than observed in Huh7-Vec and HepG2-Vec cells, respectively (Figure 2A and Figure S3A). Increased migration of Huh7-hGH and HepG2-hGH cells in comparison with Huh7-Vec and HepG2-Vec cells was also observed in the transwell assay (Figure 2B and Figure S3B). In addition, Huh7-hGH and HepG2-hGH cells exhibited significantly increased ability to invade through the Matrigel layer in the transwell invasion assay (Figure 2B and Figure S3B), and as previously reported for HepG2 cells [20].

EMT is accompanied by changes in epithelial and mesenchymal gene expression [29]. Western blot analysis revealed that forced expression of hGH downregulated the protein levels of tight junction component CLAUDIN-1 in Huh7, HepG2 and Hep3B cells (Figure 2C and Figure S3C). Concordantly, the epithelial marker E-CADHERIN was downregulated, while the extracellular matrix protein FIBRONECTIN-1 was upregulated by autocrine hGH in these HCC cells (Figure 2C and Figure S3C). We consistently observed a reduction in *CLAUDIN-1* mRNA level and promoter activity in Huh7, HepG2 and Hep3B cells with forced expression of hGH (Figure 2D,E and Figure S3D,E).

### 2.3. hGH Inhibits CLAUDIN-1 Expression Through Activation of Signal Transducer and Activator of Transcription 3 (STAT3)

**Figure 3:**
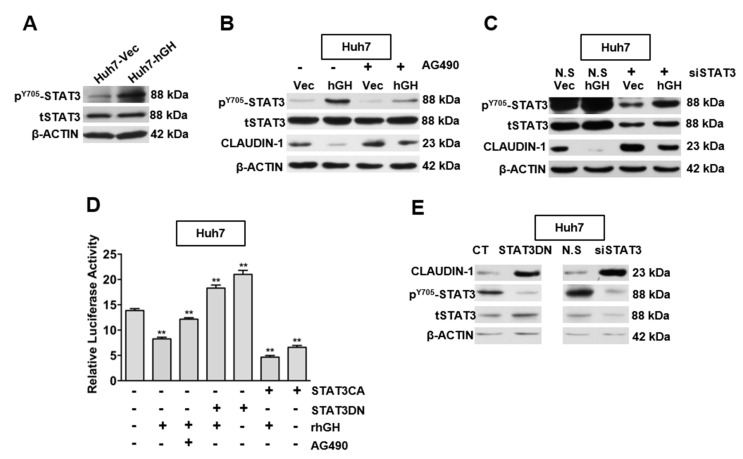
Forced expression of hGH inhibits CLAUDIN-1 expression through activated STAT3. (**A**) Levels of STAT3 Y705 phosphorylation were compared between Huh7-Vec and Huh7-hGH cell lines. β-ACTIN was used as input control; (**B**) The Y705 phosphorylation level of STAT3 and CLAUDIN-1 protein expression in Huh7-Vec/hGH cells treated with janus kinase 2 (JAK2)/STAT3 inhibitor, AG490, were determined. The Huh7-Vec/hGH cells were treated with AG490 at a concentration of 10 µM or dimethyl sulfoxide (DMSO) for 12 h and harvested for Western blot analysis; (**C**) CLAUDIN-1 expression in Huh7-Vec/hGH cells transfected with siSTAT3 was determined. Huh7-Vec/hGH cells were transfected with control nonspecific (N.S) siRNA or STAT3 siRNA. After 48 h, transfected cells were harvested and subjected to Western blot analysis for STAT3, CLAUDIN-1 and β-ACTIN expression; (**D**) hGH inhibits CLAUDIN-1 promoter activity through STAT3. Huh7 parental cells were co-transfected with CLAUDIN-1 promoter luciferase constructs, Renilla plasmids, and plasmids either encoding constitutively active STAT3 (STAT3CA) or dominant negative STAT3 (STAT3DN). At 24 h, serum-starved cells were treated with DMSO or AG490 (10 µM) for 1 h, and then these pretreated cells were stimulated by recombinant hGH (100 ng/mL) or bovine serum albumin (BSA) as control for 12 h before determining the luciferase activity of *CLAUDIN-1* promoter constructs. The statistical significance was determined in comparison to Huh7 cells without treatment. ** *p* < 0.01; (**E**) Forced expression of dominant negative STAT3 (STAT3DN) or depletion of STAT3 with siSTAT3 increased CLAUDIN-1 expression. Huh7 parental cells were transfected with control and STAT3DN plasmids (**left**), or with control siRNA and siSTAT3 (**right**). After 48 h, transfected cells were harvested and subjected to Western blot analysis for pSTAT3, tSTAT3, CLAUDIN-1 and β-ACTIN. ** *p* < 0.01.

STAT3 is a transcription factor, which is activated by cytokines and growth factors [30,31]. Previous studies have demonstrated that hGH activates janus kinase (JAK)/STAT signaling pathways in mammary and endometrial cancer cells [13,15], as well as in the HCC cell lines Bel-7404 and HepG2 [20]. Consistently, forced expression of hGH significantly increased STAT3 phosphorylation in Huh7, Hep3B and HepG2 cells without a corresponding change in total STAT3 protein levels (Figure 3A and Figure S4A).

As we observed that autocrine hGH mediated the activation of STAT3 and decreased CLAUDIN-1 expression, we further determined whether the expression of CLAUDIN-1 was inhibited by hGH-mediated STAT3 activation in HCC cells. The HCC-Vec and -hGH cells were treated with a JAK2 inhibitor (AG490) to inhibit STAT3 activation, and the resulting effect on CLAUDIN-1 expression was determined. The inhibition of STAT3 by AG490 treatment, as indicated by a decrease in STAT3 phosphorylation, restored CLAUDIN-1 expression in Huh7-hGH and Hep3B-hGH cells, to basal levels similar to those in Huh7-Vec and Hep3B-Vec cells respectively (Figure 3B and Figure S4B). Furthermore, AG490 treatment increased the basal protein level of CLAUDIN-1 in Huh7-Vec and Hep3B-Vec cells as compared with their respective dimethyl sulfoxide (DMSO)-treated counterparts (Figure 3B and Figure S4B). Similarly, the depletion of STAT3 expression by siRNA (siSTAT3) transfection restored CLAUDIN-1 protein expression to basal levels in Huh7-hGH and Hep3B-hGH cells and led to increased basal expression level of CLAUDIN-1 protein in Huh7-Vec and Hep3B-Vec cells (Figure 3C and Figure S4C). Hence, autocrine hGH mediated the decrease in CLAUDIN-1 expression through STAT3 activation.

Several studies have described a role of STAT3 as a transcriptional repressor [32,33]. Given that forced expression of hGH reduced CLAUDIN-1 protein expression through STAT3 activation in HCC cells, we investigated whether hGH regulate CLAUDIN-1 promoter activity through STAT3. Treatment with exogenous recombinant human GH (rhGH) or transfection of constitutively active STAT3 (STAT3CA) reduced CLAUDIN-1 promoter activity in Huh7 and Hep3B cells. Furthermore, the combination of rhGH treatment, together with STAT3CA transfection, achieved the highest level of inhibition on CLAUDIN-1 promoter activity as compared to either treatment alone. Conversely, AG490 treatment or dominant-negative STAT3 (STAT3DN) transfection abrogated the inhibitory effect of rhGH on CLAUDIN-1 promoter activity (Figure 3D and Figure S4D). Consistently, both the forced expression of STAT3DN and depletion of STAT3 expression by siSTAT3 increased CLAUDIN-1 protein expression in HCC cells (Figure 3E and Figure S4E).

### 2.4. Identification of a STAT3-Targeted Region within Human CLAUDIN-1 Promoter

**Figure 4:**
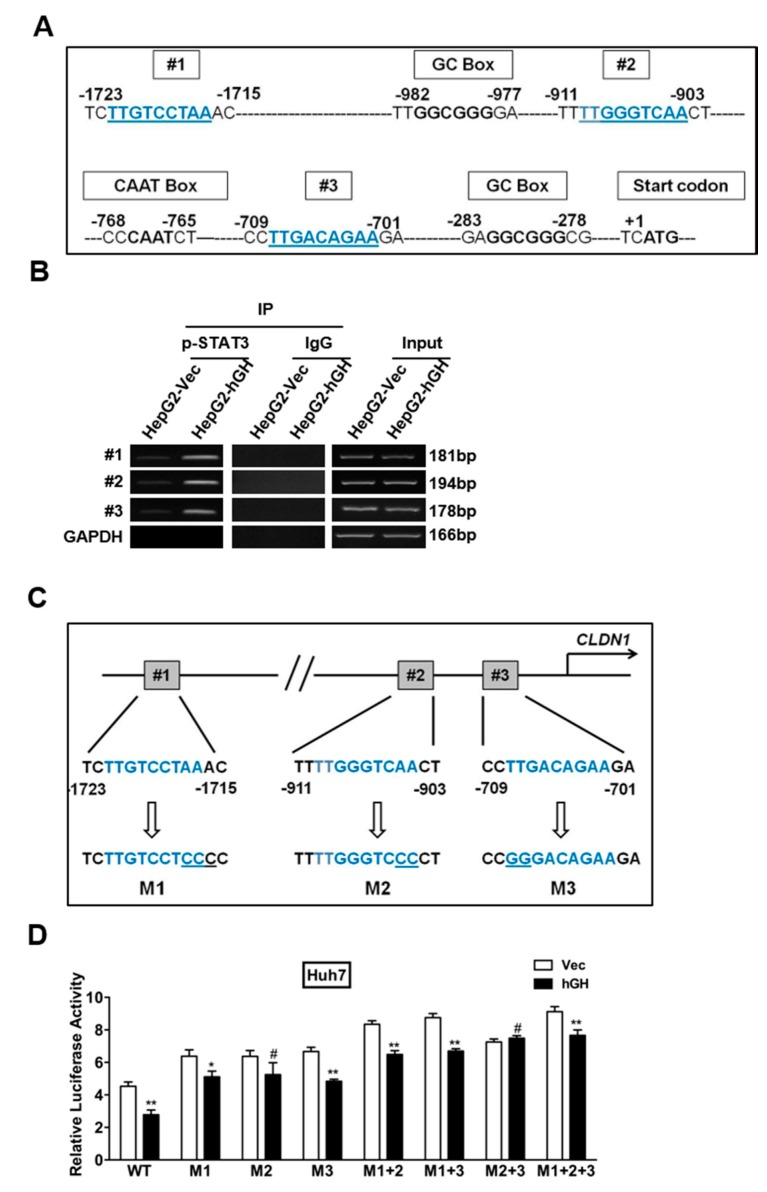
Identification of the STAT3-targeted region within the human CLAUDIN-1 promoter. (**A**) Schematic illustration of the proximal region of the human CLAUDIN-1 promoter. The human CLAUDIN-1 promoter contains three putative STAT3-binding sites shown as underlined blue letters (designated #1, #2 and #3). Two GC boxes were also identified in the proximal region of the human CLAUDIN-1 promoter as shown; (**B**) hGH enhanced direct binding of STAT3 to the three STAT3-binding sites in the CLAUDIN-1 promoter region, as revealed by chromatin immunoprecipitation (ChIP) analysis. HepG2-Vec/hGH cells were subjected to ChIP analysis using an anti-pSTAT3 antibody or a normal rabbit immunoglobulin G (IgG). DNA precipitates were PCR amplified using three pairs of primers, amplifying the three STAT3-binding sites, respectively; or primers for glyceraldehyde 3-phosphate dehydrogenase (GAPDH), used as an input control; (**C**) Mutations (M1, M2, M3) were introduced in the STAT3-binding sites in the proximal region of human CLAUDIN-1 promoter. The **top** panel shows the wildtype (WT) sequence of STAT3 binding sites within the CLAUDIN-1 promoter, while the **bottom** panel shows the mutated sequence of the STAT3 binding sites with the mutation sites being underlined; (**D**) Activity of the WT and 7 mutant luciferase reporter constructs (designated M1, M2, M3, M1 + 2, M1 + 3, M2 + 3, and M1 + 2 + 3). Huh7-Vec/hGH cells were transfected with the WT or mutant constructs, and Renilla plasmids. After 48 h, the luciferase activity was determined by normalizing against the Renilla luciferase activity. The statistical significance was determined by comparing the luciferase activity in Huh7-hGH cells to that in Huh7-Vec cells. * *p* < 0.05; ** *p* < 0.01, # no statistical significance.

We observed, thus far, that STAT3 is involved in hGH-mediated inhibition of CLAUDIN-1 gene expression, which may involve direct STAT3 modulation of the CLAUDIN-1 promoter region. Next, we determined whether the CLAUDIN-1 gene promoter contains STAT3-binding sites. As illustrated in Figure 4A, three putative STAT3-binding sites have been identified in the proximal region (1.7 kb) of the human CLAUDIN-1 promoter. A CAAT box and two GC boxes have also been identified in the CLAUDIN-1 promoter region. We therefore examined the ability of STAT3 to bind to the three putative STAT3-binding sites within the human CLAUDIN-1 promoter usingchromatin immunoprecipitation (ChIP) assay. As indicated in Figure S5A, phosphorylated STAT3 (p-STAT3) and STAT3 were observed to associate with all three putative STAT3-binding sites in the CLAUDIN-1 promoter.

We went on to investigate whether hGH modulated the ability of p-STAT3 to bind to the three putative STAT3-binding sites within the human CLAUDIN-1 promoter region. As indicated in Figure 4B, forced expression of hGH increased the binding of p-STAT3 to the three putative STAT3-binding sites in the CLAUDIN-1 promoter. In addition, we also investigated the effect of exogenous rhGH on p-STAT3 binding to the putative STAT3-binding sites in the CLAUDIN-1 promoter. Serum-deprived HepG2 parental cells were treated with 100 ng/mL rhGH for 12 h before conducting the ChIP assay. Consistent with the effect of forced expression of hGH in HepG2 stable cells, rhGH stimulation also enhanced the binding of p-STAT3 to the putative STAT3-binding sites in the CLAUDIN-1 promoter, compared with the BSA treated HepG2 cells (Figure S5B).

To analyze the functionality of the consensus STAT3 binding sites, we performed site-directed mutagenesis in these sites to generate the STAT3-binding site(s) mutated CLAUDIN-1 promoter driven luciferase reporter plasmids from the wild-type (WT) constructs. The single-site, double-site and triple-site mutated constructs were designated as M1, M2, M3, M1 + 2, M1 + 3, M2 + 3, and M1 + 2 + 3, respectively (Figure 4C). We transfected these mutated and WT CLAUDIN-1 promoter-driven luciferase reporter plasmids into HepG2 cells and observed that the STAT3-binding site mutated CLAUDIN-1 promoter exhibited significantly higher transcriptional activities than the WT CLAUDIN-1 promoter in the luciferase reporter assay (Figure S5C). In Huh7 stable cells, the promoter activity of WT CLAUDIN-1 promoter was inhibited by forced expression of hGH. However, the transcriptional activities of STAT3-binding site mutated CLAUDIN-1 promoter-driven luciferase reporter plasmids were increased compared with the WT CLAUDIN-1 promoter-driven plasmids. Meanwhile, the hGH mediated transcriptional inhibitory effect on CLAUDIN-1 promoter was abrogated by STAT3-binding site mutation (Figure 4D). A similar effect was observed in HepG2 cells treated with rhGH at a concentration of 100 ng/mL (Figure S5D).

### 2.5. Forced Expression of hGH Promotes Cancer Stem Cell (CSC)-Like Behavior of HCC Cells

**Figure 5:**
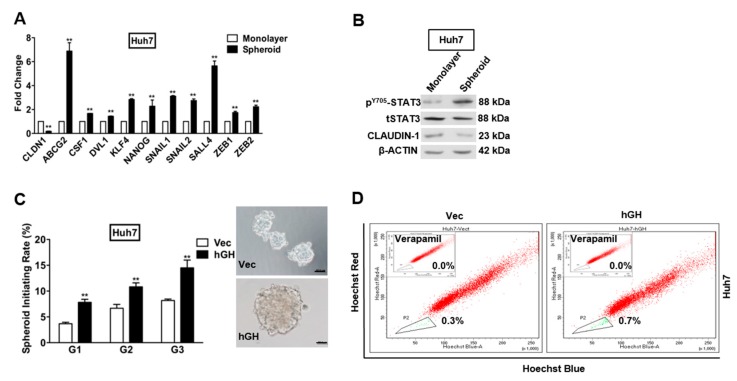
Forced expression of hGH enhances the CSC-like population in HCC cells. (**A**) Reduced expression of *CLAUDIN-1* in CSC-enriched Huh7 cells, as compared with monolayer Huh7 cells. CSC medium was used in both conditions. Spheroids were grown in ultra-low attachment plate. ** *p* < 0.01; (**B**) STAT3 activity and CLAUDIN-1 protein level in Huh7 cells grown under monolayer and spheroid culture conditions. β-ACTIN was used as input control; (**C**) Forced expression of hGH enhances spheroid formation of Huh7 cells. Huh7-Vec/hGH cells were grown under spheroid culture condition, and were sequentially cultured from first generation (G1) till third generation (G3). Images represent the first generation of spheroids formed by Huh7-Vec/hGH cells. Scale bar, 100 µm. ** *p* < 0.01; (**D**) Forced expression of hGH increases side population (SP) in Huh7 cells. Huh7-Vec/hGH cells were stained with Hoechst 33,342 with or without the ATP-binding cassette (ABC) transporter inhibitor Verapamil for 90 min. Verapamil was used to establish the baseline fluorescence of these cells. The side population within the triangle is represented by green dots, while the non-side population is represented by red dots. Flow cytometry plots indicate Hoechst Red versus Hoechst Blue.

It has been demonstrated that stem/progenitor cells and CSCs form spheroids in suspension culture [34,35]. The capacity for spheroid formation has also been identified as a CSC-like characteristic of HCC cells [35,36]. To verify the CSC-like phenotype of HCC cells in spheroids, we examined the mRNA expression of several stem cell markers in spheroids compared with cells grown as a monolayer under adherent conditions. The expression of stem cell markers in Huh7 and Hep3B spheroids were compared with the respective cells grown on monolayer in CSC medium using quantitative polymerase chain reaction (qPCR). The mRNA levels of stem cell markers, *ABCG2*, *CSF1*, *DVL1*, *KLF4*, *NANOG*, and *SALL4*, were significantly increased in spheroids compared with monolayer growth of Huh7 and Hep3B cells (Figure 5A and Figure S6A). Meanwhile, the mRNA expression of transcription factors *SNAIL1*, *SNAIL2*, *ZEB1*, and *ZEB2* was also increased in spheroid HCC cells as compared to monolayer-cultured HCC cells. In contrast, *CLAUDIN-1* mRNA level was reduced in Huh7 and Hep3B spheroids, compared with the respective monolayer grown cells (Figure 5A and Figure S6A). In addition, we also examined CLAUDIN-1 protein expression and STAT3 activity in Huh7 and Hep3B spheroids. Consistent with the qPCR results, CLAUDIN-1 protein level in Huh7 and Hep3B spheroids was significantly lower than the respective monolayer grown cells (Figure 5B and Figure S6B). Correspondingly, Huh7 and Hep3B spheroids exhibited significantly higher levels of STAT3 phosphorylation (Y705) than their respective monolayer cultured cells (Figure 5B and Figure S6B).

To determine the effect of forced expression of hGH on the CSC-like properties of HCC cells, Huh7 and Hep3B cells with forced expression of hGH were cultured under ultra-low attachment conditions. Huh7 and Hep3B cells with forced expression of hGH exhibited significantly increased growth (number) of spheroids compared with the respective -Vec cells (Figure 5C and Figure S6C). To confirm that the increase in spheroid formation represented the progeny of individual CSCs rather than the aggregation of quiescent cells, we determined the ability of primary spheroids to form secondary and tertiary spheroids. The secondary and tertiary spheroids generated from Huh7 and Hep3B cells with forced expression of hGH were significantly increased as compared with those generated from respective -Vec cells (Figure 5C and Figure S6C).

Previous studies have demonstrated that the side-population (SP) isolated from HCC cell lines exhibit CSC-like properties that are related to tumorigenesis, metastasis and therapeutic resistance [37,38]. In the current study, we determined increased mRNA expression of *ABCG2* in the CSC-enriched population from Huh7 and Hep3B cells (Figure 5A and Figure S6A), which indicates that the SP may also represent the CSC-enriched population from Huh7 and Hep3B cells. We, therefore, utilized a Hoechst 33342 efflux assay to determine the effect of autocrine hGH on Huh7 and Hep3B SPs. Flow cytometry analysis demonstrated that forced expression of hGH significantly increased the number of cells in the SP of both Huh7 and Hep3B cells compared with respective control cells (Figure 5D and Figure S6D).

### 2.6. The Autocrine hGH-Stimulated Invasive and CSC-Like Behaviors in HCC Cells Are Abrogated by Forced Expression of CLAUDIN-1

**Figure 6:**
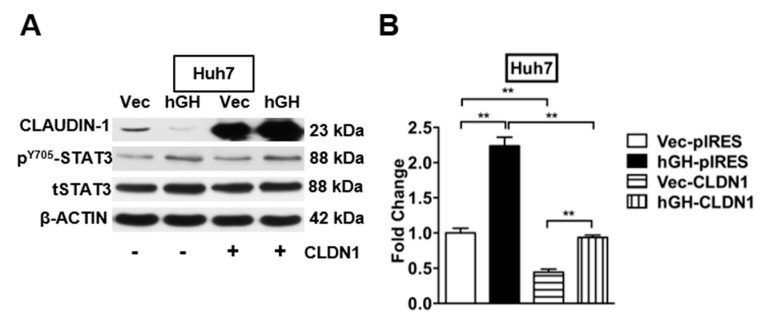

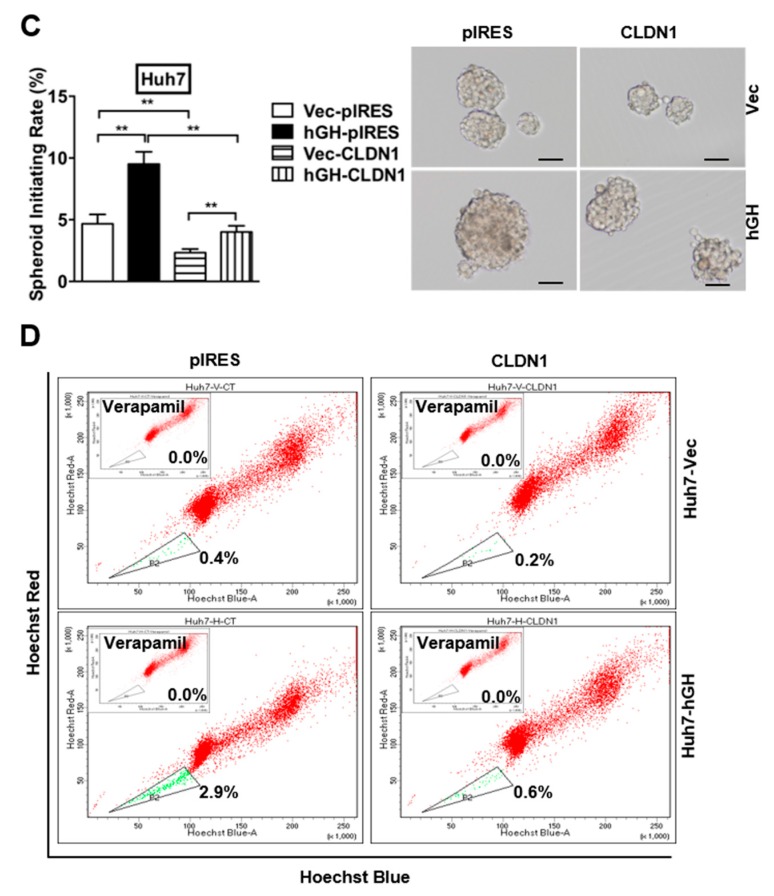
Forced expression of CLAUDIN-1 abrogated hGH promoted invasive and CSC-like properties in HCC cells. (**A**) Western blot analysis of CLAUDIN-1 expression and STAT3 activity in Huh7-Vec/hGH cell lines transfected with CLAUDIN-1 expressing plasmids (pIRES-CLDN1) or control plasmids; (**B**) Transwell assay to determine the invasive potential of Huh7-Vec/hGH cells tranfected with pIRES-CLDN1 or control plasmids. ** *p* < 0.01; (**C**) Suspension culture to determine the spheroid formation of Huh7-Vec/hGH cells transfected with pIRES-CLDN1 or control plasmids. ** *p* < 0.01; bar, 100 µm; (**D**) Hoechst 33,342 efflux assay to determine SP of Huh7-Vec/hGH cells tranfected with pIRES-CLDN1 or control plasmids. Verapamil was used to establish the baseline fluorescence of these cells. The side population within the triangle is represented by green dots, while the non-side population is represented by red dots.

Loss of CLAUDIN-1 expression correlates with worse clinical outcome and intrahepatic recurrence in poorly differentiated HCCs, which suggests that reduced CLAUDIN-1 expression may increase the metastatic and invasive potential of HCCs [27,39]. Given that forced expression of hGH inhibited the CLAUDIN-1 promoter activity, and decreased the expression of CLAUDIN-1 gene in HCC cells at both mRNA and protein levels, we proposed that hGH stimulates invasion by decreasing CLAUDIN-1 expression. Huh7- and Hep3B-VEC/hGH cells were transiently transfected with CLAUDIN-1 expression plasmid pIRES-CLDN1, and the expression of CLAUDIN-1 was confirmed by Western blot assay (Figure 6A and Figure S7A). In the transwell invasion assay, autocrine hGH stimulated invasive of Huh7-hGH and Hep3B-hGH cells was abrogated by CLAUDIN-1 overexpression, and concordantly overexpression of CLAUDIN-1 further attenuated the basal invasive potential of Huh7-Vec and Hep3B-Vec cells (Figure 6B and Figure S7B).

Decreased CLAUDIN-1 expression in a CSC-enriched population of HCC cells indicates a potential role of CLAUDIN-1 in maintaining the CSC-like population (Figure 5A,B and Figure S6A,B). To determine whether hGH-induced CSC-like behavior was mediated by CLAUDIN-1 in HCC cells, pIRES-CLDN1 was transiently transfected into Huh7-hGH and Hep3B-hGH and their respective -Vec cells. Overexpression of CLAUDIN-1 abrogated the stimulatory effect of autocrine hGH on spheroid formation in both Huh7 and Hep3B cells (Figure 6C and Figure S7C). In addition, forced expression of CLAUDIN-1 also largely abrogated the hGH-induced increase in SP in Huh7-hGH and Hep3B-hGH cells (Figure 6D and Figure S7D).

### 2.7. Depletion of CLAUDIN-1 Enhances Invasive and CSC-Like Behaviors of HCC Cells

**Figure 7:**
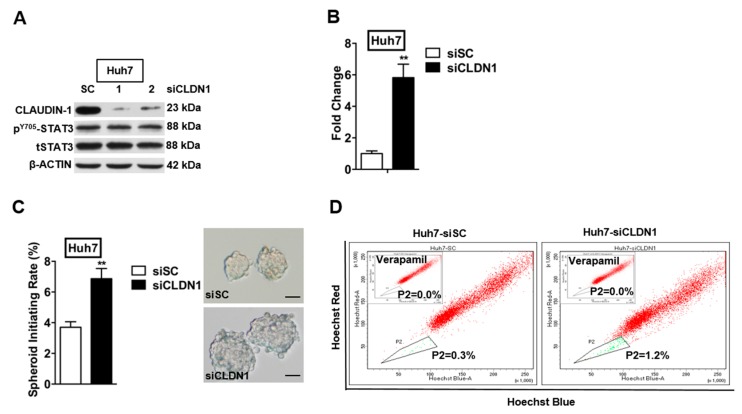
Depletion of CLAUDIN-1 enhances invasive and CSC-like properties of HCC cells. (**A**) Western blot analysis for CLAUDIN-1 expression and STAT3 activity in Huh7 cells transfected with CLAUDIN-1 specific siRNAs or scrambled siRNA; (**B**) Standard Transwell assay to assess the invasive potential of Huh7 cells transfected with CLAUDIN-1 specific siRNA, or scrambled siRNA as control. ** *p* < 0.01; (**C**) Suspension culture to determine the effect of CLAUDIN-1 depletion on self-renewal properties of CSC-like population in Huh7 cells. The Huh7 cells were transfected with CLAUDIN-1 specific siRNAs or scrambled siRNAs. The transfected cells were cultured with CSC medium in ultra-low attachment. ** *p* < 0.01; bar, 100 µm; (**D**) Hoechst 33,342 efflux assay to determine the effect of CLAUDIN-1 depletion on SP in Huh7 cells. The Huh7 cells were transfected with CLAUDIN-1 specific siRNAs or scrambled siRNA. The transfected cells were stained with Hoechst 33,342 in absence or presence of Verapamil, and then analyzed by flow cytometry. Verapamil was used to establish the baseline fluorescence of these cells. The side population within the triangle is represented by green dots, while the non-side population is represented by red dots.

To further study the suppressive potential of CLAUDIN-1, we depleted CLAUDIN-1 expression by small interfering RNAs (siRNA) specifically targeting CLAUDIN-1 (siCLDN1-1 and siCLDN1-2) in Huh7 and Hep3B parental cells, and this was confirmed by Western blot (Figure 7A and Figure S8A). We selected siCLDN1-1 for further functional studies. The transwell invasion assay demonstrated that Huh7-siCLDN1 cells exhibited dramatically increased invasive capacity as compared with the control cells (Figure 7B). A similar trend was observed in Hep3B cells with depleted CLAUDIN-1 expression (Figure S8B). Concordantly, depletion of CLAUDIN-1 expression resulted in increased spheroid formation in suspension culture and increased SP in both Huh7-siCLDN1 and Hep3B-siCLDN1 cells, compared with the respective control cells (Figure 7C,D and Figure S8C,D).

### 2.8. Depletion of CLAUDIN-1 Attenuates Cell Proliferation without Affecting Cell Apoptosis

**Figure 8:**
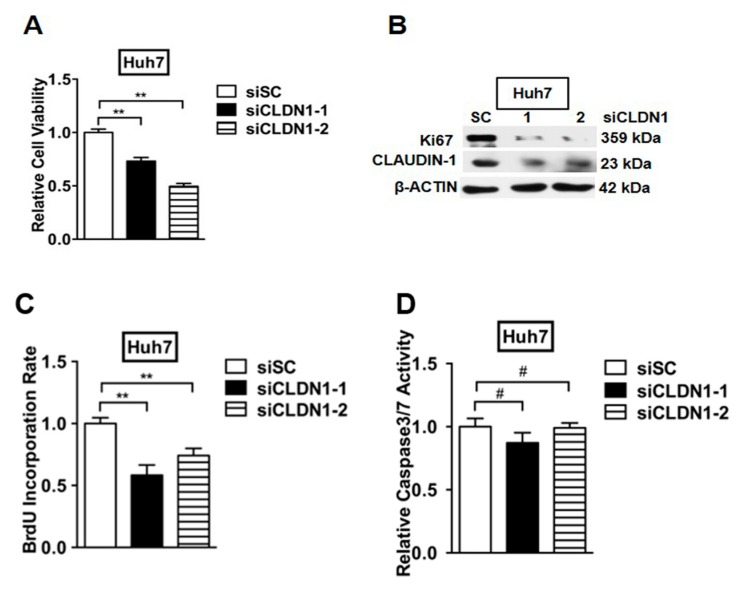
CLAUDIN-1 depletion decreases cell proliferation of HCC cells without modulating cell survival. (**A**) Cell viability of Huh7 cells with CLAUDIN-1 depletion. The Huh7 cells were transfected with CLAUDIN-1 specific siRNAs or scrambled siRNAs. After 48 h, cell viability was determined by MTT assay. ** *p* < 0.01; (**B**) Western blot analysis for Ki67, a cell proliferating marker in Huh7 cells transfected with CLAUDIN-1 specific siRNAs or scrambled siRNAs; (**C**) Cell cycle progression of Huh7 cells transfected with CLAUDIN-1 specific siRNAs or scrambled siRNAs was assessed by BrdU incorporation. ** *p* < 0.01; (**D**) Huh7 cells were transfected with CLAUDIN-1 specific siRNAs or scrambled siRNAs for 24 h, and cell apoptosis was subsequently assessed by measuring caspase 3/7 activity. # no statistical significance.

To further examine the effect of CLAUDIN-1 on HCC cell viability, Huh7 and Hep3B cells were transfected with CLAUDIN-1 specific siRNAs or scrambled siRNA as control. The viability of transfected cells was determined by 3-(4,5-dimethylthazol-2yl)-2,5-diphenyltetrazolium bromide (MTT) assay after 48 h of transfection. Depletion of CLAUDIN-1 significantly reduced the viability of Huh7 and Hep3B cells (Figure 8A and Figure S9A).

We next determined whether reduction of cell viability by depleting CLAUDIN-1 was attributed to decreased cell proliferation. Western blot analysis demonstrated that the depletion of CLAUDIN-1 significantly reduced the protein level of cell proliferation marker Ki67 compared with respective control cells (Figure 8B and Figure S9B). We also examined whether depletion of CLAUDIN-1 reduced the S-phase entry of HCC cells. BrdU incorporation was decreased in Huh7 and Hep3B cells with CLAUDIN-1 depletion (Figure 8C and Figure S9C). However, depletion of CLAUDIN-1 did not modulate HCC cell apoptosis, as indicated by the absence of a significant change in caspase 3/7 activities, compared with the respective control cell lines (Figure 8D and Figure S9D).

## 3. Discussion

GH transgenic mice with elevated circulating GH exhibited increased rates of spontaneous [16] and carcinogen diethylnitrosamine (DEN)-induced HCC [17]. An aberrant increase in circulating hGH levels has been observed in patients with chronic liver disease [40] and hepatocellular carcinoma [41]. Higher levels of hGHR were detected at the mRNA and protein levels by in situ hybridization and IHC in HCC tissues as compared to normal liver tissues [19]. Our clinical investigation has also determined that increased expression of *hGH* mRNA correlates with poor clinical outcome in HCC patients, indicative of the functional effects of autocrine or tumor derived hGH in HCC [20]. We have also demonstrated herein that increased autocrine hGH expression functionally enhances oncogenicity and invasive potential of HCC cells in vitro, which is concordant with previous studies that demonstrated the role of hGH in oncogenic transformation and phenotypic conversion of mammary, endometrial and hepatocellular carcinoma cells [11,12,28]. hGH has been reported to primarily utilize hGHR but has also been shown to bind to hPRLR to activate JAK2 [42,43]. The hGH/JAK2/STAT3 signaling transduction pathway has been identified as a critical signaling pathway in breast cancer and HCC [20,44]. Furthermore, autocrine hGH-induced proliferative and anti-apoptotic effects have been shown to be mediated by the JAK2/STAT3 signaling pathway in endometrial carcinoma [15]. In addition, hGH has been shown to activate STAT3 in hepatoma cell line HepG2 [45] and fibrosarcoma cell line [15,46]. STAT3 regulates liver regeneration by stimulating hepatic cell proliferation and survival [47]. The STAT3 activated signaling pathways increase the expression of various target genes involved in cell proliferation and survival, including *CYCLIN D1*, *c-MYC*, *SURVIVIN*, and *BCL2L1* [48,49]. The increased expression and constitutive activation of STAT3 have been detected in the vast majority of human HCC patient samples [50,51]. Consistent with our previous studies [15,20], we have shown herein that the hGH-stimulated STAT3 activity is critical for promoting the proliferation and survival of HCC cells for HCC progression.

EMT is a postulated key step for tumor metastasis whereby the integrity of epithelial cell layer is disrupted with the loss of cell polarity and cell-cell contacts, and cell motility is increased to facilitate the infiltration of cancer cells into surrounding tissues [29]. Herein we have demonstrated that autocrine hGH promotes HCC cell migration and invasion. Changes in HCC cell gene expression stimulated by autocrine hGH was consistent with the altered in vitro cell behaviors. Autocrine hGH increased the expression of mesenchymal cell marker FIBRONECTIN and reduced the expression of epithelial cell marker E-CADHERIN, concordant with our previous studies in mammary, endometrial and hepatocellular carcinoma [11,12]. In addition, we also observed the down-regulation of CLAUDIN-1 gene, a tight junction constituent. Reduced CLAUDIN-1 expression has been reported in poorly differentiated HCCs and associated with portal invasion [27]. Moreover, attenuated expression of CLAUDIN-1 is significantly associated with a poor overall survival rate, suggesting that CLAUDIN-1 is an independent prognostic factor in HCC [27]. Consistent with the clinicopathological studies, we demonstrated that CLAUDIN-1 functions as a tumor suppressor gene in HCC cells. Forced expression of CLAUDIN-1 abrogated autocrine hGH-induced cell invasion in HCC cells. Moreover, depletion of CLAUDIN-1 expression significantly increased cell invasion of HCC cells in vitro. In conjunction with increased invasion, the proliferation of HCC cells was inhibited by depletion of CLAUDIN-1. We also demonstrated that CLAUDIN-1 did not modulate HCC cell survival in monolayer culture consistent with a previous study described in breast cancer [52]. However, CLAUDIN-1 has been shown to promote breast cancer cell apoptosis in suspension culture [52], suggesting that loss of CLAUDIN-1 may protect detached cancer cells from anoikis in blood circulation and facilitate tumor metastatic progression. In contrast to our observation that CLAUDIN-1 inhibits EMT, Lee et al. have demonstrated that CLAUDIN-1 induces EMT phenotypic conversion of normal human liver cells, thereby promoting invasiveness through increased expression of SNAIL and SLUG [53,54]. The contradictory observations may represent distinctive roles of CLAUDIN-1 at different stages of HCC development. CLAUDIN-1 expression may facilitate HCC initiation, and then be inhibited during the aggressive progression of HCC. The differential roles of a gene in the initiation and progression of HCC have previously been reported [55]. An example is that telomere dysfunction promotes HCC initiation but suppresses HCC progression, and telomerase function was observed to be re-activated during HCC progression [56].

The existence of CSCs in HCC has been posited as a key contributor to drug resistance, tumor metastasis and relapse [57]. Integrative comparative genomic studies have revealed that a subtype of HCC, which is associated with poor clinical outcome, is derived from normal hepatic stem/progenitor cells [58]. Elevated expression of the GHR gene in embryonic and tissue-specific stem cells has been determined through analysis of gene expression profiles, indicating the functional effects of GH/GHR signal pathways in stem cells [34,59]. In addition, GHR-positive mammary stem/progenitor cells exhibited increased self-renewal capability in response to paracrine GH stimulation [60]. Concordant with the current study, we have previously observed that autocrine hGH promotes the CSC-like properties in triple negative breast cancer cells [14]. Activation of STAT3 and expression of stem cell markers NANOG and OCT4 have been reported in liver stem/progenitor cells [61]. Aberrant activation of STAT3 has been demonstrated to maintain the CSC population of liver cancer through increased expression of NANOG [57]. This supports our observation that the hGH-stimulated increase in CSC-like behavior in HCC cells depends on STAT3-mediated repression of CLAUDIN-1.

The reasons for poor prognosis in late stage HCC includes invasive dissemination and tumor relapse, which can be ascribed to EMT and CSC-like characteristics. The link between EMT and CSC-like behavior has been well-established in various cancers, including HCC [62,63,64,65]. The transcription factors TWIST-1 and SNAIL that promote EMT progression, concordantly induce the CSC phenotype [62,65]. In our study, the CSC-enriched population in spheroid colonies exhibited increased expression of stem cell markers, as well as transcription factors that regulate EMT transformation, indicating that the CSCs in HCC may play a vital role in tumor metastatic dissemination and relapse. A previous study has determined that, in addition to promoting EMT, TGF-β induced snail1 stimulates CSC phenotype through the NANOG transcription in liver cancer [65]. We have found that the expression of the epithelial marker CLAUDIN-1 is repressed in the CSC-enriched population, suggestive that the loss of CLAUDIN-1 is the mediator of both autocrine hGH-stimulated invasiveness and CSC-like phenotypes in HCC cells. However, previous studies in ovarian cancer have demonstrated that CLAUDIN-1 promotes proliferation and self-renewal of ovarian CSCs [66,67]. The contradicting studies may indicate tissue-specific effects of CLAUDIN-1 in cancer progression. Our study identified autocrine hGH as a dual effector promoting EMT and CSC-like properties in HCC cells. This suggests that the hGH/hGHR signaling is a potential therapeutic target to prevent HCC metastasis and relapse [20].

Furthermore, we have elucidated a novel mechanism of STAT3-mediated transcriptional repression of CLAUDIN-1 in HCC cells. The expression of CLAUDIN-1 is regulated by diverse mechanisms. Transcription factors SNAIL and SLUG inhibit CLAUDIN-1 expression through binding to the two E-box elements in the 5′ proximal region of CLAUDIN-1 promoter [68]. Similarly, the transcription of other epithelial markers namely E-CADHERIN and OCCLUDIN, have been demonstrated to be repressed through transcription factors binding to their promoter E-box elements, thereby triggering EMT [69,70]. STAT3 plays a vital role in inducing EMT during progression of various types of cancer [71]. Constitutive STAT3 activation has been observed in HCC tissues, and is significantly associated with HCC invasion and metastasis [51]. Although STAT3 is widely accepted to promote gene transcription [72], we have shown here that STAT3 acts as a transcriptional repressor to inhibit CLAUDIN-1 expression in HCC cells. Previous studies have demonstrated that STAT3 inhibits the transcription of inducible nitric oxide synthase (iNOS) and interleukin-8 (IL-8) through direct interaction with nuclear factor κ-light-chain-enhancer of activated B cells (NF-κB) [32,33,73]. Putative NF-κB elements in the 5′ proximal region of CLAUDIN-1 promoter have been identified [74]. However, there is no evidence yet that NF-κB is involved in the regulation of CLAUDIN-1 expression. Moreover, we could not exclude the possibility of indirect regulation of STAT3 on CLAUDIN-1 expression, as STAT3 has been demonstrated to regulate the expression and activation of SNAIL-1 and SLUG [75,76], which are known repressors of CLAUDIN-1 expression [68]. Further study is required to determine the mechanism of STAT3 inhibition of CLAUDIN-1 expression.

## 4. Materials and Methods

### 4.1. Cell Lines and Cell Transfection

The liver cancer cell lines Huh7, HepG2, and Hep3B, were obtained from the Institute of Virology, Chinese Academy of Medical Sciences (Beijing, China). All of these cell lines were cultured using Dulbecco’s Modified Eagle Medium (DMEM) medium supplemented with 10% heat-inactivated fetal bovine serum (FBS). The hGH expression plasmid pcDNA3.1-hGH was generated as previously described [11]. Huh7, HepG2, and Hep3B cell lines were stably transfected with pcDNA3.1-hGH plasmid (designated Huh7-hGH, HepG2-hGH, and Hep3B-hGH) or empty pcDNA3.1 plasmid (designated Huh7-Vec, HepG2-Vec, and Hep3B-Vec).

### 4.2. In Vitro Cell Function Assays

Cell proliferation, apoptosis, and oncogenicity were determined by total cell number, cell viability, BrdU incorporation, Caspase 3/7 activity, soft agar, and three-dimensional growth assays as previously described [11,15,77] with minor modifications. For total cell number, Huh7-Vec, Huh7-hGH, HepG2-Vec, and HepG2-hGH cell lines were seeded into six-well plates at 10^4^ cells per well with complete medium. The cells were trypsinized and counted using a hemocytometer every two days over a period of eight days. For cell viability, cells were seeded into 96-well plates at a density of 5000 cells per well in complete medium. The 3-(4,5-dimethylthazol-2yl)-2,5-diphenyltetrazolium bromide (MTT) assay was conducted to determine cell viability as described previously [11]. For cell cycle, cells were seeded into six-well plates. Cell cycle was determined by 5-Bromo-2-deoxyuridine (BrdU) incorporation. For cell apoptosis, cells seeded into six-well plates were serum starved for 24 h; then cell apoptosis was determined by measuring caspase 3/7 activities using the Caspase-Glo^®^ 3/7 Assay Kit (Promega, Madison, WI, USA). For anchorage-independent growth, cells (10^4^ cells per well) were seeded with 0.3% agar gel into 24-well plates pre-coated with 0.7% agar gel. For three-dimensional growth, 10^3^ cells were plated in 10% fetal bovine serum (FBS) medium supplemented with 4% Matrigel (BD Biosciences, San Jose, CA, USA) in 96-well plates. Medium containing 4% Matrigel was changed every 2 day over 10 day. Cell viability was determined using MTT Standard Transwell assays were performed as previously described [77] to determine cell migration and invasion. The migration and invasion assays were performed at 24 and 48 h, respectively.

### 4.3. Spheroid Formation Assay

The spheroid formation assay was performed as previously described [34] with some modifications. Monolayer cells were trypsinized and resuspended in Dulbecco’s modified Eagle’s medium F12 (Invitrogen, Carlsbad, CA, USA) supplemented with 20 ng/mL recombinant human epidermal growth factor (EGF) (Sigma-Aldrich, St. Louis, MO, USA), 20 ng/mL recombinant basic fibroblast growth factor (bFGF) (BD Bioscience), 2% B27 (Invitrogen), 5 μg/mL bovine insulin (Sigma-Aldrich), and 1% penicillin-streptomycin (Invitrogen). The suspensions were passed through a 40 μm cell strainer (BD Bioscience) to generate single cell suspensions. The single-cell suspensions were seeded in ultra-low attachment 6-well plates (Corning, Tewksbury, MA, USA) at 10^4^ cells per mL. The subsequent passages were grown at the same density, and cell colonies were counted as described previously [34].

### 4.4. Side Population

The side population analysis was performed according to the protocol described by Goodell et al. with minor modifications [78]. Cells harvested by trypsinization and centrifuged were incubated in DMEM medium containing 2% FBS and Hoechst 33,342 (5 μg/mL) for 90 min at 37 °C with or without the ATP-binding cassette (ABC) transporter inhibitor, verapamil (50 μM). Cells were then collected by centrifugation and resuspended in 1 mL cold phosphate-buffered saline (PBS). The samples were analyzed by flow cytometry (FACS LSR II, BD Bioscience).

### 4.5. Polymerase Chain Reaction (PCR) and Western Blot Analysis

Semiquantitative RT-PCR and quantitative PCR were performed aspreviously described [11]. The sequences of the primers used are listed in Tables S1 and S2.

Western blot analysis was carried out using the following antibodies: rabbit hGH polyclonal antibody (National Hormone and Peptide Program), mouse β-ACTIN monoclonal antibody (Santa Cruz, Dallas, TX, USA), rabbit CLAUDIN-1 monoclonal antibody (Abcam, Cambridge, UK), rabbit FIBRONECTIN-1 monoclonal antibody (Abcam), mouse E-CADHERIN monoclonal antibody (Abcam), mouse N-CADHERIN monoclonal antibody (Abcam), rabbit p^Y705^-STAT3 monoclonal antibody (Cell Signaling, Danvers, MA, USA), mouse total STAT3 monoclonal antibody (Cell Signaling).

### 4.6. Chromatin Immunoprecipitation

The chromatin immunoprecipitation was carried out using EZ-Magna ChIP^TM^ Kit (Millipore, Billerica, MA, USA), with anti-rabbit monoclonal p^Y705^-STAT3 antibody and three pairs of primers to amplify the three putative STAT3-binding sites in the CLAUDIN-1 promoter region as follows: #1, 5′-CAACACGAACATGGTCTTGTCCTA-3′ and 5′-TCCCTGGATGCCTTTCATGTAG-3′; #2, 5′-GCTGCTCTAAGACATAACTGCTGTG-3′ and 5′-ACGCACGGAGCATATCCAGTA-3′; #3, 5′-GTACTGGATATGCTCCGTGCGT-3′ and 5′-CCCCTTCTTTCCTCTCTCTGCT-3′.

### 4.7. STAT3-Binding Site Mutated CLAUDIN-1 Promoter-Driven Luciferase Reporter Constructs

The wild type CLAUDIN-1 promoter-driven luciferase reporter construct was a kind gift from Yoshiaki Ito [26]. Based on the wild-type constructs, STAT3-binding site mutated CLAUDIN-1 promoter-driven luciferase reporter constructs were generated using QuikChange II XL Site-Directed Mutagenesis Kit (Agilent Technologies, Santa Clara, CA, USA) as illustrated in Figure 4C.

### 4.8. Construction of CLAUDIN-1 Expression Plasmids

The *CLAUDIN-1* gene was amplified from the cDNA of normal hepatic cell line LO2 using the following primers: 5′-GGATCCATGGCCAACGCGGGGCTG-3′ and 5′-GCGGCCGCTCACACGTAGTCTTTCCCGCTGGAA-3′. The amplified DNA segment was cloned into the pIRESneo3 vector (Clontech Laboratories, Mountain View, CA, USA) between the BamHI and NotI restriction sites. The construct was verified using DNA sequencing.

### 4.9. Luciferase Reporter Assay

Cells were transfected using Lipofectamine 2000 (Invitrogen) according to the protocol from manufacturer. The luciferase activities of the reporter constructs were determined using the Dual-Luciferase Reporter Assay System (Promega).

### 4.10. Statistical Analysis

GraphPad Prism 5 (GraphPad Software, Inc., La Jolla, CA, USA) was used to generate graphical presentations and for statistical analysis. All experiments were performed at least three times and a single representative figure was shown. Numerical data were presented as mean ± standard error mean (SEM). Data were analyzed using an unpaired two-tailed *t*-test with *p* < 0.05 being considered as statistically significant.

## 5. Conclusions

In summary, we have demonstrated that autocrine hGH increased invasive and CSC-like properties in HCC cells. Furthermore, we have determined that autocrine hGH stimulated invasive and CSC-like properties in HCC are mediated through STAT3-dependent inhibition of CLAUDIN-1 expression and identified CLAUDIN-1 as a novel STAT3-repressed downstream target in HCC cells. Inhibition of hGH/hGHR signaling pathways could be considered as a potential paradigm to limit progression and relapse of HCC.

## Supplementary Materials

Supplementary materials can be found at www.mdpi.com/1422-0067/18/6/1274/s1.

## Abbreviations

hGH	Human growth hormone
HCC	Hepatocellular carcinoma
EMT	Epithelial to mesenchymal transition
CSCs	cancer stem cells
