# Supplementary material for: Autocrine Human Growth Hormone Promotes Invasive and Cancer Stem Cell-Like Behavior of Hepatocellular Carcinoma Cells by STAT3 Dependent Inhibition of CLAUDIN-1 Expression
Source: Int J Mol Sci. 2017 Jun 15;18(6):1274. doi: 10.3390/ijms18061274 (PMC5486096; doi:10.3390/ijms18061274)
Supplement: Supplementary file 1 [file ijms-18-01274-s001.pdf]

Supplemental

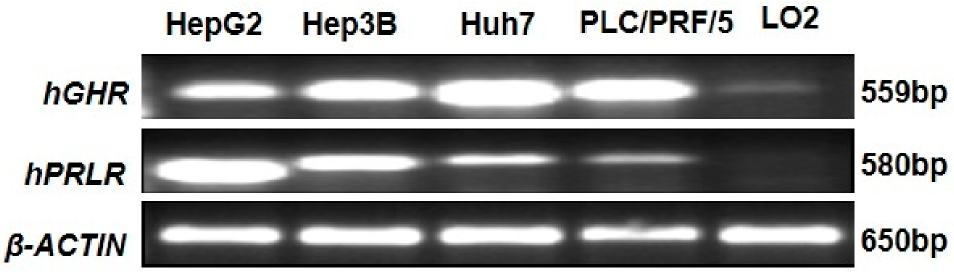

Fig. S1. Expressions of hGHR and hPRLR were determined by RT-PCR in human liver cancer cell lines and a normal liver cell line LO2. *β-ACTIN* was used as input control.

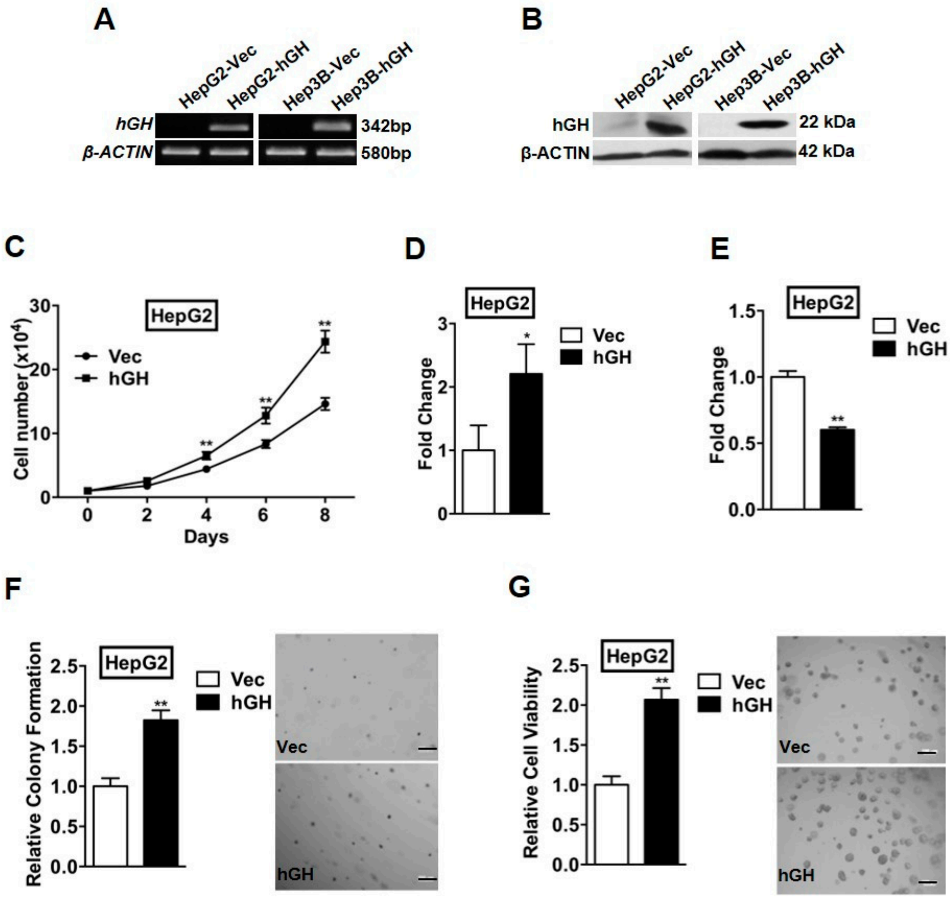

Fig. S2. Forced expression of hGH promotes cell proliferation, cell survival, and anchorage-independent growth in human HCC cells. HepG2 and Hep3B cells were stably transfected with an expression vector containing the *hGH* gene (designated HepG2-hGH and Hep3B-hGH) or pcDNA vector alone (HepG2-Vec and Hep3B-Vec). The expression level of *hGH* in HepG2-Vec/hGH and Hep3B-Vec/hGH cell lines was determined by RT-PCR (A) and western blot (B). β-ACTIN was used as input control. C, Growth of HepG2-Vec and HepG2-hGH cells were determined by total cell number in medium supplemented with 10% FBS. *, $p<0.05$; **, $p<0.01$. D, Effect of forced expression of hGH on cell cycle progression was assessed by BrdU incorporation in medium supplemented with 10% FBS. E, Effect of forced expression of hGH on cell apoptosis was determined by measuring caspase 3/7 activity of HepG2-Vec and HepG2-hGH cell lines after 24h serum-deprivation. *, $p<0.05$. F, the oncogenic transformation of HepG2 cells by forced expression of hGH was assessed by soft agar colony formation in 10% FBS medium. Cell colonies were visualized by MTT staining. *Bar*, 200 μm; **, $p<0.01$. G, Effect of forced expression of hGH on the growth of HepG2 cells on growth factor-reduced Matrigel™. HepG2-Vec/hGH cells were seeded in 4% Matrigel™ into 96-well plates pre-coated with Matrigel™. Cell viability was measured using MTT assay after 7 days. *Bar*, 200 μm; **, $p<0.01$.

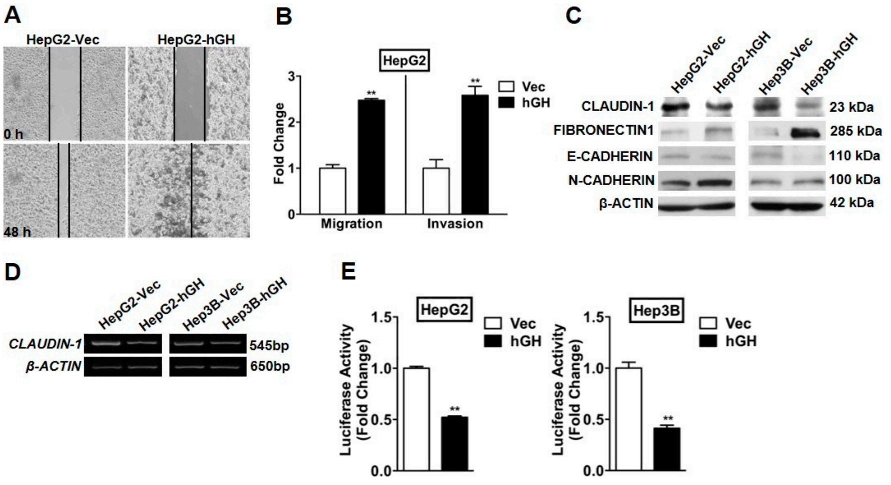

Fig. S3. Forced expression of hGH promotes cell motility and invasive phenotype in human HCC cells. A, Cell motility of HepG2-Vec/hGH cell lines was assessed by wound-healing assay under ×100 magnification. B, The effect of forced expression of hGH on HepG2 cell migration and invasion was determined by standard Transwell assay. C, Western blot analysis of the expression of epithelial and mesenchymal markers in HepG2-Vec/hGH and Hep3B cell lines. D, the mRNA expression of CLAUDIN-1 gene in HepG2-Vec/hGH and Hep3B-Vec/hGH cell lines was assessed by RT-PCR. E, The CLAUDIN-1 promoter activity in HepG2-Vec/hGH and Hep3B-Vec/hGH cell lines was assessed by luciferase reporter assay.

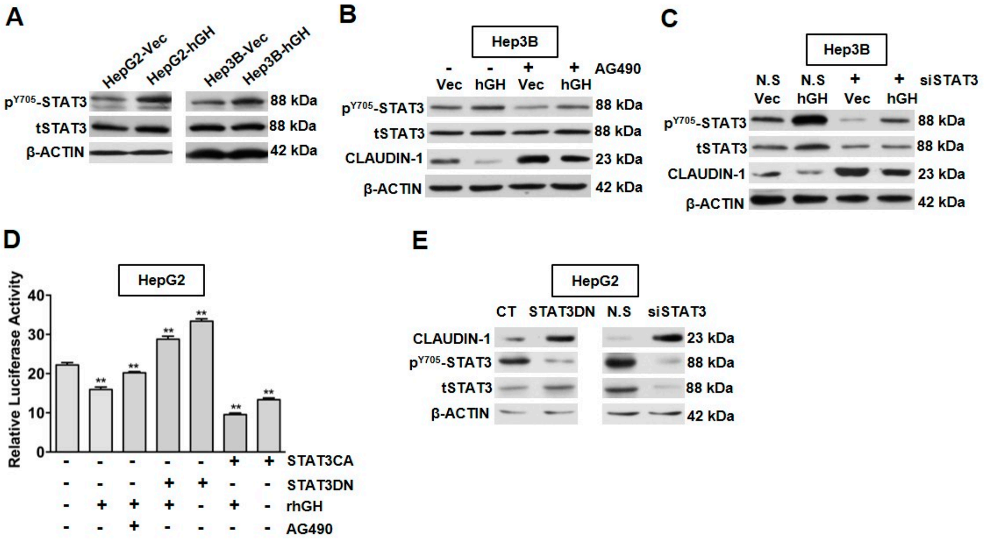

Fig. S4. Forced expression of hGH inhibits claudin-1 expression through activated STAT3. A, Levels of STAT3 Y705 phosphorylation were compared in HepG2-Vec/hGH and Hep3B-Vec/hGH cell lines. β-ACTIN was used as input control. B, The Y705 phosphorylation level of STAT3 and claudin-1 protein expression in Hep3B-Vec/hGH treated with JAK2/STAT3 inhibitor AG490 cells were determined. The Hep3B-Vec/hGH cells were treated with AG490 at a concentration of 10 μM or DMSO for 12h before collected for western blot analysis. C, claudin-1 expression in Hep3B-Vec/hGH cells transfected with siSTAT3 was determined. Hep3B-Vec/hGH cells were transfected with control nonspecific interfering RNA (N.S) and STAT3 small interfering RNA. After 48h, transfected cells were harvested and subjected to western blot analysis for STAT3, claudin-1 and β-ACTIN expression. D, hGH inhibits *CLAUDIN-1* promoter activity through STAT3. HepG2 parental cells were co-transfected with *CLAUDIN-1* promoter luciferase constructs, Renilla plasmids, and indicated plasmids (STAT3CA and STAT3DN). At 24h, serum-starved cells were treated with DMSO or AG490 (10 μM) for 1h, and then these pretreated cells were stimulated by BSA or recombinant hGH (100ng/ml) for 12h before determining the luciferase activity of *CLAUDIN-1* promoter constructs. The statistical significance was determined by comparing with Huh7 cells without treatment. **, $p<0.01$. E, Forced expression of domain negative STAT3 (STAT3DN) and STAT3 small interfering RNA (siSTAT3) increased claudin-1 expression. HepG2 parental cells were transfected with control and STAT3DN plasmids (*left*), of transfected with control siRNA and siSTAT3 (*right*). After 48h, transfected cells were harvested and subjected to western blot analysis for pSTAT3, tSTAT3, claudin-1 and β-ACTIN.

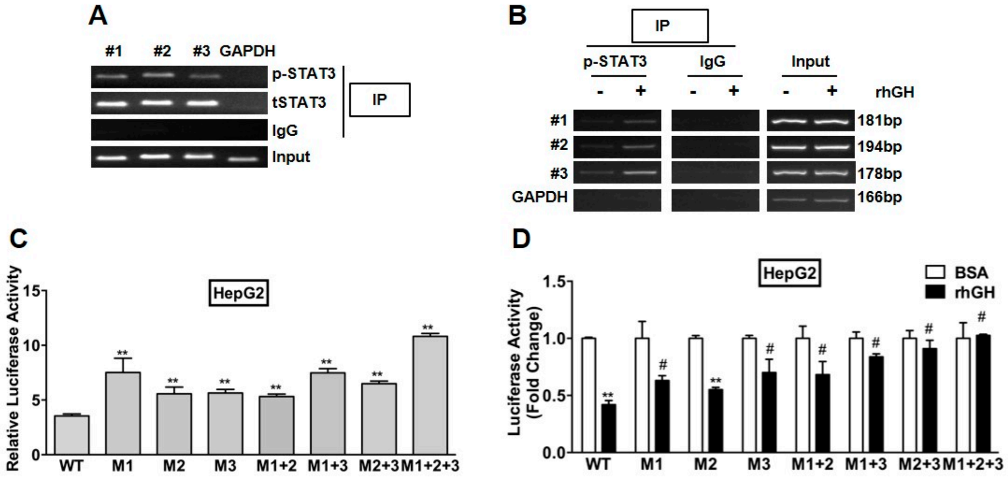

Fig. S5. Identification of STAT3-targeted region within human CLAUDIN-1 promoter. STAT3 directly binds to the three STAT3-binding sites in the CLAUDIN-1 promoter region, as revealed by ChIP analysis. HepG2 parental cells were subjected to ChIP analysis using an anti-pSTAT3 antibody, anti-total STAT3 antibody, or a normal rabbit IgG. DNA precipitates were PCR amplified using three pairs of primers, amplifying the three STAT3-binding sites, respectively; or primers for GAPDH, used as an internal control. B, hGH enhanced direct binding of STAT3 to the three STAT3-binding sites in the CLAUDIN-1 promoter region, as revealed by ChIP analysis. Serum-starved HepG2 parental cells were treated with rhGH (100ng/ml) or BSA for 12h before subjected to ChIP analysis using an anti-pSTAT3 antibody or a normal rabbit IgG. DNA precipitates were PCR amplified using three pairs of primers, amplifying the three STAT3-binding sites, respectively; or primers for GAPDH, used as an internal control. C, mutant CLAUDIN-1 promoter constructs exhibited higher transcriptional activity. The WT and mutant CLAUDIN-1 promoter constructs were transfected into HepG2 parental cells. Ranilla plasmids were used as control for transfecting efficiency. After 48h, the luciferase activity of these constructs was assessed. The statistical significance was determined by comparing the luciferase activity of WT constructs. D, Activity of the WT and the 7 mutant luciferase reporter constructs in response to rhGH treatment. HepG2 parental cells transfected with the WT or mutant constructs were serum-starved, and then treated with BSA or 100ng/ml rhGH for 12h. The luciferase activity was determined by normalizing against the Renilla luciferase. The statistical significance was determined by comparing the relative luciferase activity of HepG2 cells treated with rhGH to the HepG2 cells treated with BSA. *, $p<0.05$; **, $p<0.01$, #, no statistic significance.

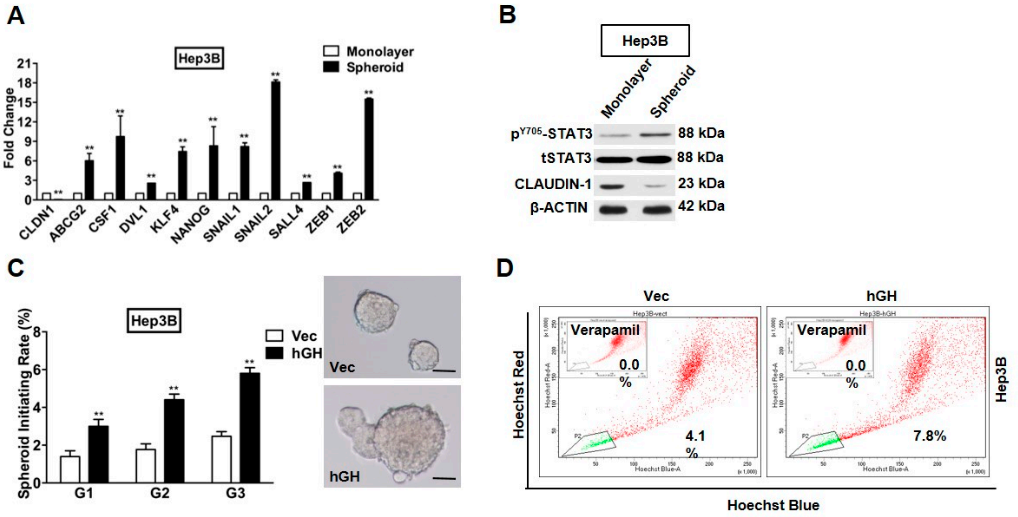

Fig. S6. Forced expression of hGH enhances CSC-like population in HCC cells. A, Reduced expression of CLAUDIN-1 in CSC-enriched Hep3B cells, as compared with monolayer Hep3B cells. Cancer stem cell medium was used in both conditions. Spheroids were grown in ultra-low attachment plate. **, $p<0.01$. B, STAT3 activity and claudin-1 protein level in Hep3B cells grown under monolayer and spheroidic conditions. β-ACTIN was used as input control. C, Forced expression of hGH enhances spheroid formation of Hep3B cells. Hep3B-Vec/hGH cells were grown under spheroidic condition, and were sequentially cultured from first generation (G1) till third generation (G3). Images represent the first generation of spheroids formed by Hep3B-Vec/hGH cells. Bar, 100 μm. **, $p<0.01$. D, forced expression of hGH increases Side Population (SP) in Hep3B cells. Hep3B-Vec/hGH cells were stained with Hoechst 33342 with or without the ABC transporter inhibitor Verapamil for 90min. Verapamil was used to establish the baseline fluorescence of these cells. Flow cytometry plots indicate Hoechst Red *versus* Hoechst Blue.

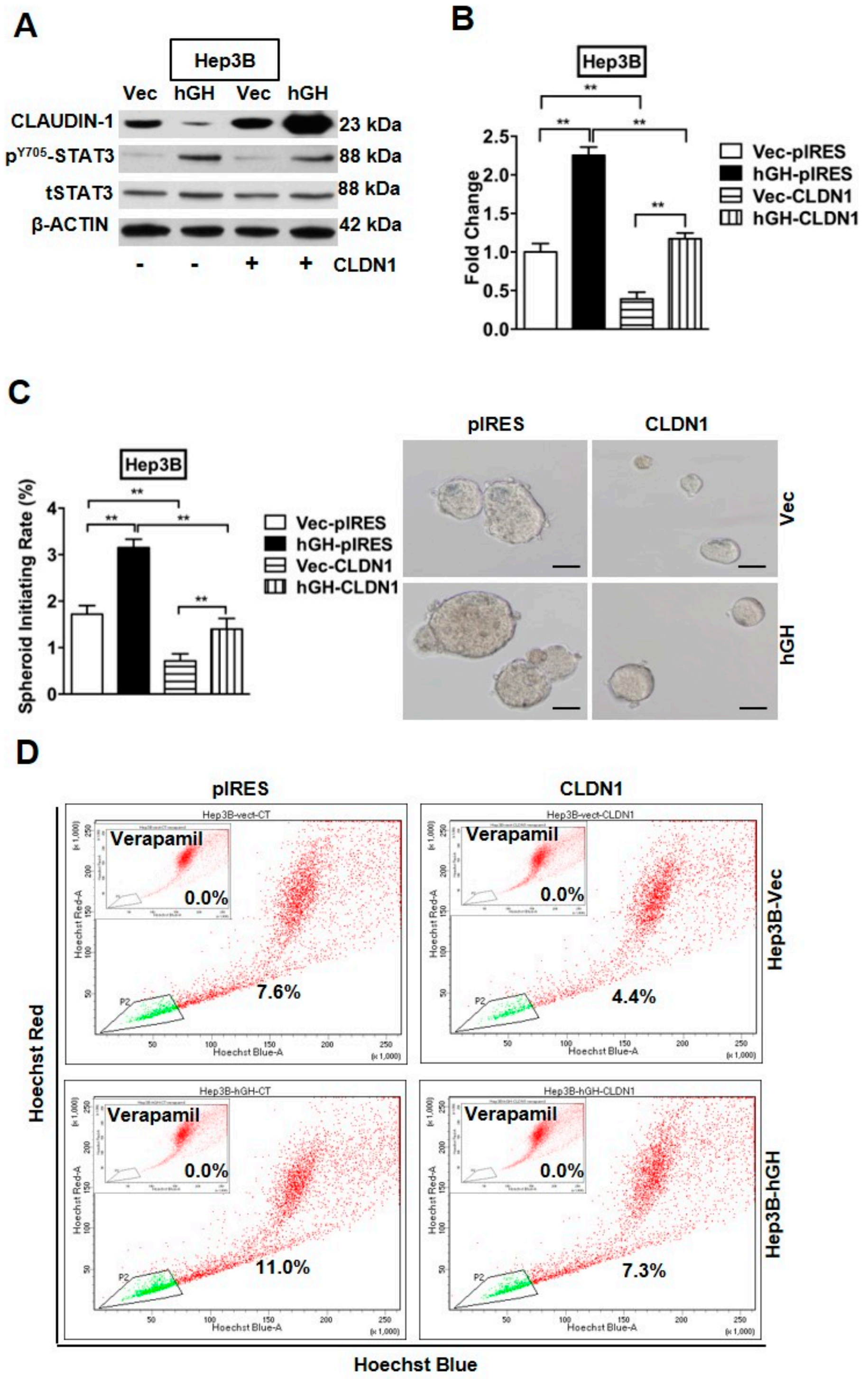

Fig. S7. Forced expression of CLAUDIN-1 abrogated hGH promoted invasive and CSC-like properties in HCC cells. A, Western blot analysis of claudin-1 expression and STAT3 phosphorylation in Hep3B-Vec/hGH cell lines transfected with claudin-1 expressing plasmids (pIRES-CLDN1) or control plasmids. B, The standard Transwell assay to determine the invasive potential of Hep3B-Vec/hGH cells tranfected with pIRES-CLDN1 or control plasmids. **, p<0.01. C, Suspension culture to determine the spheroid formation of Hep3B-Vec/hGH cells transfected with pIRES-CLDN1 or control plasmids. **, p<0.01; *bar*, 100 μm. D, Hoechst 33342 efflux assay to determine SP of Hep3B-Vec/hGH cells tranfected with pIRES-CLDN1 or control plasmids. **, p<0.01.

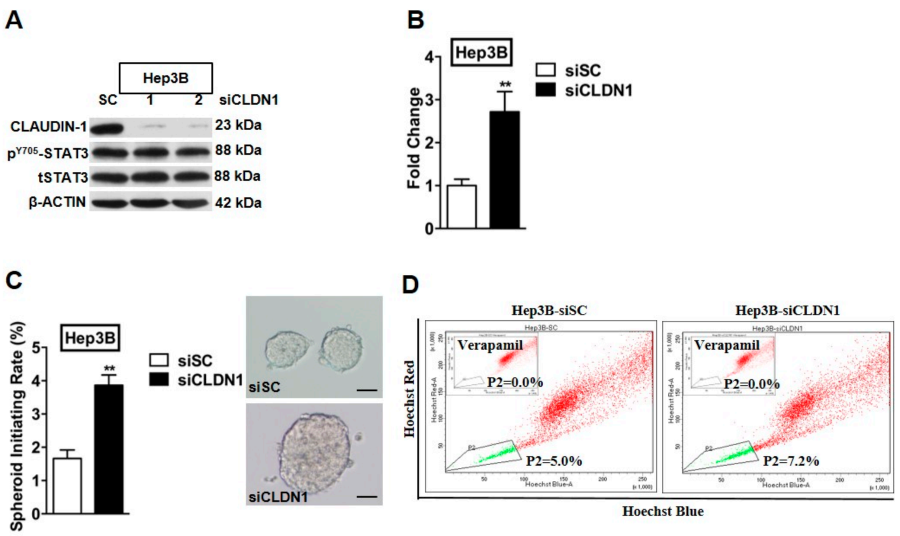

Fig. S8. Depletion of CLAUDIN-1 enhances invasive and CSC-like properties of HCC cells. A, Western blot analysis of CLAUDIN-1 expression and STAT3 phosphorylation in Hep3B cells transfected with CLAUDIN-1 specific siRNAs or scrambled siRNA. B, Standard Transwell assay to assess the invasive potential of Hep3B cells transfected with CLAUDIN-1 specific siRNA, or scrambled siRNA as control. **, $p<0.01$. C, Suspension culture to determine the effect of CLAUDIN-1 depletion on self-renew of CSC-like population in Hep3B cells. The Hep3B cells were transfected with CLAUDIN-1 specific siRNAs or scrambled siRNA. The transfected cells were cultured with CSC medium in ultra-low attachement. **, $p<0.01$; bar, 100 μm. D, Hoechst 33342 efflux assay to determine the effect of CLAUDIN-1 depletion on SP in Hep3B cells. The Hep3B cells were transfected with CLAUDIN-1 specific siRNAs or scrambled siRNA. The transfected cells were stained with Hoechst 33342 in the absence or presence of Verapamil, and then analyzed by flow cytometry.

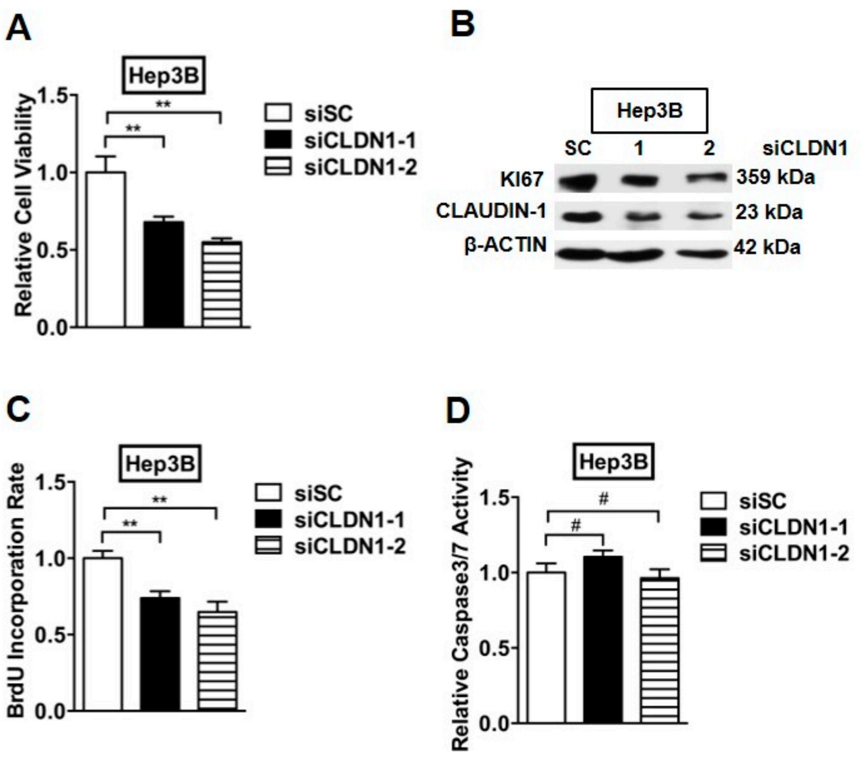

Fig. S9. Effects of CLAUDIN-1 depletion on cell proliferation and survival in HCC cells. A, Cell viability of Hep3B cells with CLAUDIN-1 depletion. The Hep3B cells were transfected with CLAUDIN-1 specific siRNAs or scrambled siRNAs. After 48h, cell viability was determined by MTT assay. **, p<0.01. B, Western blot analysis for ki67, a cell proliferating marker in Hep3B cells transfected with CLAUDIN-1 specific siRNAs or scrambled siRNAs. C, Cell cycle progression of Huh7 cells transfected with CLAUDIN-1 specific siRNAs or scrambled siRNAs was assessed by BrdU incorporation. **, p<0.01. D, Hep3B cells transfected with CLAUDIN-1 specific siRNAs or scrambled siRNAs for 24h, and cell apoptosis was assessed by measuring caspase 3/7 activity. #, no statistical significance.
